# Oxygenation influences xylose fermentation and gene expression in the yeast genera *Spathaspora* and *Scheffersomyces*

**DOI:** 10.1186/s13068-024-02467-8

**Published:** 2024-02-07

**Authors:** Katharina O. Barros, Megan Mader, David J. Krause, Jasmyn Pangilinan, Bill Andreopoulos, Anna Lipzen, Stephen J. Mondo, Igor V. Grigoriev, Carlos A. Rosa, Trey K. Sato, Chris Todd Hittinger

**Affiliations:** 1 DOE Great Lakes Bioenergy Research Center, University of Wisconsin-Madison, Madison, WI USA; 2 Laboratory of Genetics, Wisconsin Energy Institute, J. F. Crow Institute for the Study of Evolution, Center for Genomic Science Innovation, University of Wisconsin-Madison, Madison, WI USA; 3 U.S. Department of Energy Joint Genome Institute, Lawrence Berkeley National Laboratory, Berkeley, CA USA; 4 Department of Computer Science, San Jose State University, One Washington Square, San Jose, CA USA; 5 Department of Agricultural Biology, Colorado State University, Fort Collins, CO USA; 6 Environmental Genomics and Systems Biology Division, Lawrence Berkeley National Laboratory, Berkeley, CA USA; 7 Plant and Microbial Department, University of California Berkeley, Berkeley, CA USA; 8 Departamento de Microbiologia, ICB, C.P. 486, Universidade Federal de Minas Gerais, Belo Horizonte, Brazil

**Keywords:** Serinales (CUG-Ser1 clade), Xylose fermentation, Ethanol, Xylitol, Xylose reductase, Xylitol dehydrogenase, Cofactors, Gene expression, Aeration

## Abstract

**Background:**

Cost-effective production of biofuels from lignocellulose requires the fermentation of d-xylose. Many yeast species within and closely related to the genera *Spathaspora* and *Scheffersomyces* (both of the order Serinales) natively assimilate and ferment xylose. Other species consume xylose inefficiently, leading to extracellular accumulation of xylitol. Xylitol excretion is thought to be due to the different cofactor requirements of the first two steps of xylose metabolism. Xylose reductase (XR) generally uses NADPH to reduce xylose to xylitol, while xylitol dehydrogenase (XDH) generally uses NAD^+^ to oxidize xylitol to xylulose, creating an imbalanced redox pathway. This imbalance is thought to be particularly consequential in hypoxic or anoxic environments.

**Results:**

We screened the growth of xylose-fermenting yeast species in high and moderate aeration and identified both ethanol producers and xylitol producers. Selected species were further characterized for their XR and XDH cofactor preferences by enzyme assays and gene expression patterns by RNA-Seq. Our data revealed that xylose metabolism is more redox balanced in some species, but it is strongly affected by oxygen levels. Under high aeration, most species switched from ethanol production to xylitol accumulation, despite the availability of ample oxygen to accept electrons from NADH. This switch was followed by decreases in enzyme activity and the expression of genes related to xylose metabolism, suggesting that bottlenecks in xylose fermentation are not always due to cofactor preferences. Finally, we expressed *XYL* genes from multiple *Scheffersomyces* species in a strain of *Saccharomyces cerevisiae*. Recombinant *S. cerevisiae* expressing *XYL1* from *Scheffersomyces xylosifermentans*, which encodes an XR without a cofactor preference, showed improved anaerobic growth on xylose as the primary carbon source compared to *S. cerevisiae* strain expressing *XYL* genes from *Scheffersomyces stipitis*.

**Conclusion:**

Collectively, our data do not support the hypothesis that xylitol accumulation occurs primarily due to differences in cofactor preferences between xylose reductase and xylitol dehydrogenase; instead, gene expression plays a major role in response to oxygen levels. We have also identified the yeast *Sc. xylosifermentans* as a potential source for genes that can be engineered into *S. cerevisiae* to improve xylose fermentation and biofuel production.

**Supplementary Information:**

The online version contains supplementary material available at 10.1186/s13068-024-02467-8.

## Introduction

Xylose is the most abundant pentose that comprises hemicellulose in plants; therefore, robust microorganisms that can ferment this sugar are required for profitable biofuel production from lignocellulosic materials [1, 2]. The budding yeast *Saccharomyces cerevisiae* is widely used in biotechnological applications and is one of the most understood model microorganisms [3, 4]. However, *S. cerevisiae* lacks certain traits that limit its usefulness in lignocellulosic biofuel production, prompting some to investigate other yeast species as alternative biocatalysts. For example, *S. cerevisiae* does not have the ability to ferment xylose [5, 6], while several non-conventional yeast species do so remarkably well [7].

Species belonging to the genera *Spathaspora* and *Scheffersomyces* (order Serinales under a recently proposed taxonomy [8], formerly the CUG-Ser1 major clade [9]) are known for their association with insects and their habitats, such as decomposing wood, and by their natural ability to assimilate and/or ferment xylose [10–12]. *Spathaspora passalidarum, Scheffersomyces stipitis* (syn. *Pichia stipitis*)*, Scheffersomyces segobiensis, Scheffersomyces shehatae, Scheffersomyces coipomoensis*, and *Scheffersomyces ergatensis* were the first members assigned to their respective clades [13, 14]. *Sp. passalidarum* and *Sc. stipitis* have also been used as sources of genes that have been engineered in *S. cerevisiae* to confer the ability to ferment xylose [15–17]. These non-conventional yeasts harbor three genes that encode enzymes for xylose metabolism: *XYL1,* which encodes xylose reductase (XR) for the reduction of xylose to xylitol; *XYL2*, which encodes xylitol dehydrogenase (XDH) for the conversion of xylitol to xylulose; and *XYL3*, which encodes xylulokinase (XK) for the phosphorylation of xylulose to xylulose-5-phosphate [18]. The genomes of most studied xylose-fermenting species contain an XR that preferentially utilizes NADPH as its primary cofactor and has lower affinity for NADH, while the XDH is strictly NAD^+^ dependent [19–21]. A commonly articulated hypothesis is that different cofactor preferences lead to an imbalance during xylose catabolism; specifically, xylitol accumulates in anoxic environments or oxygen-limited conditions because little or no NAD^+^ can be regenerated without sufficient oxygen to act as a terminal electron acceptor [15, 22].

Some yeast species have adopted genetic mechanisms that manage cofactor imbalances during xylose catabolism. Unlike *Sc. stipitis*, *Sp. passalidarum* bears two homologs of *XYL1*, which are named *XYL1.1* and *XYL1.2*. The first one encodes an XR with NADPH affinity, while the enzyme encoded by the second homolog prefers NADH over NADPH [15]. Interestingly, xylose metabolism in this species is driven toward ethanol production, instead of xylitol accumulation, even at low aeration [23, 24]. Indeed, mutating the XR to prefer NADH over NADPH or inserting *Sp. passalidarum XYL1.2* increases the ethanol productivity and alleviates cofactor imbalance in *S. cerevisiae* [15, 25, 26]. In addition to enzyme specificity, expression of the *XYL* genes, particularly *XYL2*, in *S. cerevisiae* has been observed to impact xylose utilization, as higher expression led to more efficient xylose fermentation [16, 27]. The XR:XDH expression ratios have also been suggested to impact xylitol accumulation and the yield of ethanol produced [28]. Beyond the first steps of xylose metabolism, the overexpression of genes related to the non-oxidative phase of the pentose phosphate pathway (PPP) has also been shown to positively influence the growth rate on xylulose [29]. Although these factors seem to be important for xylose metabolism, genetically modified *S. cerevisiae* strains still cannot ferment xylose at rates comparable to glucose [5, 30, 31]. The presence of a complete, integrated *XYL* pathway alone is not sufficient for xylose assimilation and/or fermentation by *S. cerevisiae* [27, 32], suggesting that other genetic and regulatory mechanisms are required for efficient xylose metabolism. For this reason, investigations of native xylose-fermenting yeast species may provide novel strategies to overcome this problem in *S. cerevisiae*, which has impeded bioenergy research for decades.

The overall aims of this study are to understand how some species belonging to the genera *Spathaspora* and *Scheffersomyces* can efficiently ferment xylose into ethanol, why other species incompletely catabolize the pentose and accumulate xylitol, and how oxygenation can impact these outcomes. Although several members of these genera have been described in the taxonomic literature in recent years, little physiological information is available for most of them, and most earlier studies have been confined to *Sp. passalidarum* and *Sc. stipitis*. Our data show that xylose metabolism for species from the order Serinales is highly plastic and that oxygenation has a broad effect on the gene expression of xylolytic and interacting pathways.

## Results

### *Scheffersomyces* and *Spathaspora* switch ethanol production to xylitol or glycerol production during respiration

Species belonging to the order Serinales, particularly members of the genera *Scheffersomyces* and *Spathaspora* [33], are known to harbor the uncommon ability to ferment xylose. We first determined the quantities of metabolites that these species produced under moderate oxygen-limited growth conditions with xylose as the primary carbon source (see Methods). Ethanol and xylitol were the major metabolites produced by *Spathaspora* and *Scheffersomyces* species during xylose fermentation, while glycerol was produced less frequently. We refer to ethanol and xylitol producers for species that primarily excreted these metabolites (based on yields) under moderate aeration in unbaffled shake flasks (SF). The titers, productivity rates, and yields related to the consumption of d-xylose and the production of biomass, ethanol, and xylitol under moderate and high aeration conditions are summarized in Additional file 1.

**Fig. 1:**
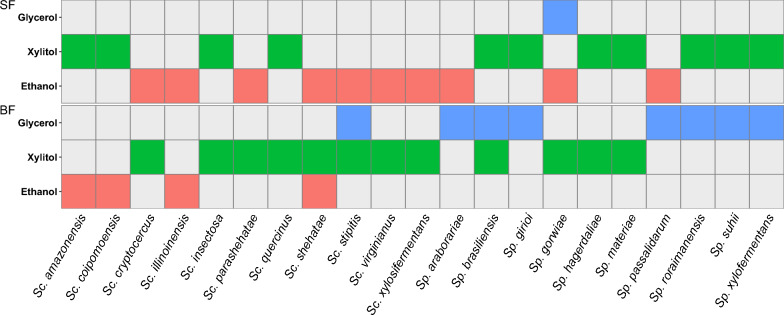
Yeast species from the order Serinales generate different metabolic end-products based on oxygen levels. Production of ethanol, xylitol, and/or glycerol by species of *Scheffersomyces* and *Spathaspora* genera under moderate (shake flask—SF) and high (baffled flask—BF) aeration conditions. Colored squares: metabolite(s) mainly produced by each species based on yields. Gray squares: the species produced low yields or did not produce the metabolite. Complete data are in Additional file 1

The species that produced ethanol under oxygen-limited conditions (41.7% of the yeasts tested) were *Scheffersomyces xylosifermentans*, *Sc. parashehatae*, *Sc. virginianus*, *Sc. shehatae*, *Sc. stipitis*, *Sc. illinoinensis*, *Sc. cryptocercus*, *Spathaspora arborariae*, *Sp. passalidarum*, and *Sp. gorwiae*. Remarkably, *Sc. xylosifermentans*, *Sc. parashehatae*, and *Sp. passalidarum* consumed all the xylose and reached maximum titers and yields of ethanol within 24 h of fermentation. After this time, the species consumed the ethanol. The xylitol producers (45.8%) included *Scheffersomyces coipomoensis*, *Sc. insectosa*, *Sc. amazonensis*, *Sc. quercinus*, *Sp. brasiliensis*, *Sp. suhii*, *Sp. roraimanensis*, *Sp. girioi*, *Sp. hagerdaliae*, *Sp. xylofermentans*, and *Candida (Spathaspora) materiae*. Except for the production of ethanol by *Sc. amazonensis*, *Sc. coipomoensis*, and *C. materiae*, the yield and productivity of xylitol surpassed ethanol production, and these species accumulated the highest titers of xylitol compared to other xylitol producers. Out of the 24 species of *Spathaspora* and *Scheffersomyces* tested, three were not capable of fermenting xylose and are not visualized in Fig. 1: *Sc. spartinae* and *Sc. gosingicus* showed minimal growth and modest consumption of sugar with no production of any metabolite examined, while *Sc. ergatensis* did not grow.

**Fig. 2:**
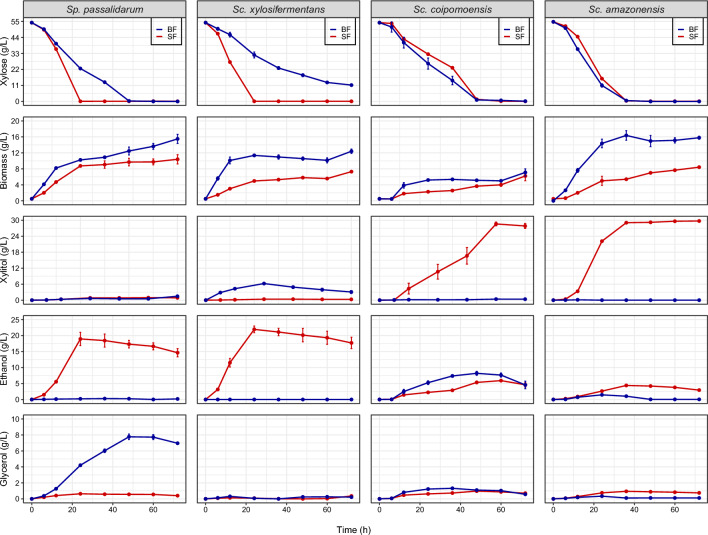
*Scheffersomyces xylosifermentans* and *Spathaspora passalidarum* ferment xylose into ethanol at high yield and titer. Consumption of xylose and production of biomass, ethanol, xylitol, and glycerol by *Sc. xylosifermentans*, *Sp. passalidarum*, *Scheffersomyces amazonensis*, and *Scheffersomyces coipomoensis* under moderate (shake flask—SF) and high (baffled flask—BF) aeration conditions. Error bars indicate the standard deviation from the three biological replicates

To verify the impact of oxygenation on xylose usage by *Spathaspora* and *Scheffersomyces* species, we also tested the species in baffled flasks (BF). This condition increases the volumetric mass transfer coefficient of oxygen (kLa), which is a parameter that determines the rate at which a gaseous compound can transfer between the gas and liquid phases (Li et al., 2013). Using an oxygen meter, we determined that the dissolved oxygen level in BF (4.74 mg L^−1^) was nearly double the oxygen present in SF (2.63 mg L^−1^). Although respiration dominated fermentation under high aeration, multiple species still fermented, while several species produced different primary metabolites in BF (Fig. 1). *Sc. xylosifermentans*, which presented high consumption of xylose and ethanol yields in SF, accumulated xylitol under high aeration and it did not consume xylose completely in 72 h. *Sp. passalidarum* produced glycerol, instead of ethanol, and the consumption of xylose was delayed in BF. For some species, especially xylitol producers, oxygen increased the rate of xylose utilization, and xylose was consumed faster under high aeration than moderate oxygen-limited conditions. Figure 2 shows the distinction between ethanol producers (*Sp. passalidarum* and *Sc. xylosifermentans*) and xylitol producers (*Sc. coipomoensis* and *Sc. amazonensis*). In contrast to the former two species, *Sc. coipomoensis* and *Sc. amazonensis* had slightly enhanced xylose consumption and produced ethanol, instead of xylitol, under high aeration. Even so, *Sc. coipomoensis* and *Sc. amazonensis* produced more biomass under these conditions, which limited their ethanol production relative to the ethanol producers in SF.

For all the species studied here, the increased oxygen levels in BF favored the production of cell biomass. With greater oxygen availability in BF, we expected that the metabolism would be directed mainly toward cellular respiration. In general, the yeasts produced higher end-product (ethanol/xylitol) titers in SF compared to BF. Despite the xylitol production by the ethanol producers, this result suggests that most of the carbon was converted into carbon dioxide (CO_2_) in BF.

### Most species with multiple *XYL1* homologs accumulate xylitol under moderate and high aeration

Recently, a study reported the presence of multiple homologs of *XYL1* in *Candida intermedia*, which harbors three *XYL1* genes, one of which (called *XYL1.2*) encodes an XR with higher affinity for NADH [19]. Like the *XYL1.2* from *Sp. passalidarum*, *C. intermedia XYL1.2* displays dual cofactor specificity [15, 19]. A search for homologs across the order Serinales showed that *Candida blattae*, *Meyerozyma carpophila*, *Me. guilliermondii,* and *Me. caribbica* also have more than one *XYL1* homologs. To determine whether these species ferment xylose as efficiently as *Sp. passalidarum* and to investigate the influence of multiple *XYL1* homologs on xylose metabolism, we tested them, as well as *C. intermedia,* during growth in YPX (1% yeast extract, 2% peptone, 5% xylose) under moderate and high aeration. Xylose was completely consumed by these strains under high aeration, while a significant amount of xylose remained unused under moderate conditions. These results suggested that xylose utilization by these closely related species was highly dependent on oxygen. All the species outside of the *Scheffersomyces* and *Spathaspora* clades accumulated xylitol in both conditions (Additional file 2). Although the yield of xylitol surpassed the yield of ethanol produced during moderate aeration by *C. intermedia*, it was the only species able to achieve a substantial titer of ethanol. Its fermentative capability may be due to the presence of an enzyme that favors the use of NADH. However, the cofactor preferences of the enzymes encoded by the homologs of *XYL1* from the other species are not known.

### Xylose reductase from *Scheffersomyces xylosifermentans* has dual cofactor affinity

**Fig. 3:**
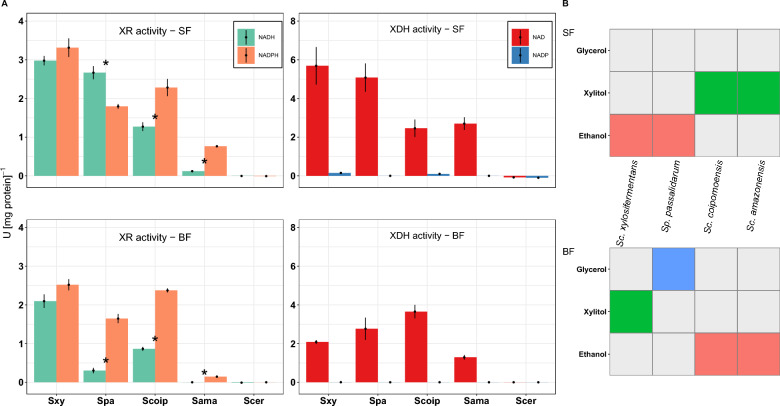
Xylose reductase from *Scheffersomyces xylosifermentans* lacks a cofactor preference. **A** XR and XDH activities under moderate aeration and high aeration conditions expressed in units (U) per mg protein [U (mg protein)^−1^]. **B** Production of ethanol, xylitol, and/or glycerol by the species tested for XR and XDH activities (reproduced from Fig. 1). Colored squares: metabolite(s) mainly produced by each species based on yields. Gray squares: the species produced low yields or did not produce the metabolite. Spa—*Spathaspora passalidarum*, Sxy—*Scheffersomyces xylosifermentans*, Scoip—*Scheffersomyces coipomoensis*, Sama—*Scheffersomyces amazonensis*, and Scer—*Saccharomyces cerevisiae* (negative control). Error bars indicate the standard deviation from the three biological replicates. Asterisks denote significant differences between the activities on NADH and NADPH for each species (*P* < 0.05)

Xylitol accumulation during metabolic conversion of xylose has been proposed to result from imbalanced cofactor usage by XR and XDH. To determine the cofactor preference of the enzymes in the first steps of xylose metabolism, we measured the specific activity of XR and XDH from ethanol-producing *Sp. passalidarum* and *Sc. xylosifermentans*, as well as from xylitol-producing *Sc. amazonensis* and *Sc. coipomoensis*, in SF and BF (Fig. 3A). The rationale for selecting *Sc. xylosifermentans*, *Sc. coipomoensis*, and *Sc. amazonensis* was that they harbor a single homolog of the *XYL1* gene, and they exhibited distinct phenotypes (ethanol versus xylitol production) under the tested conditions (Fig. 3B). In SF, XR of *Sc. coipomoensis* and *Sc. amazonensis* utilized NADH, but we observed greater XR activities with NADPH (*P* < 0.05) (Fig. 3A). On the contrary, there were no significant differences in *Sc. xylosifermentans* XR activities between the two cofactors. This result is interesting because, among these species, *Sc. xylosifermentans* showed the highest consumption of xylose in SF. In BF, the cofactor preferences of the enzymes were not significantly different, which was expected given the presence of a single homolog of *XYL1*.

Previous research showed that XR activities from *Sp. passalidarum* utilized both NADH and NADPH with higher activity for NADH under moderate oxygen-limited conditions and higher activity for NADPH in high aeration [15, 23]. Our results were consistent with those previous observations: *Sp. passalidarum* XR significantly preferred NADH over NADPH (*P* < 0.05) in SF and significantly preferred NADPH (*P* < 0.05) in BF. This switch in cofactor preference could have been enabled by the differential regulation of the two distinct *XYL1* homologs. This species also has two homologs of *XYL2* [34], but there is no data available in the literature showing the preference of XDH from each homolog. Our results revealed that XDH was strictly NAD^+^ dependent not only for *Sp. passalidarum*, but for all the four species tested. Low activities with NADP^+^ occurred for *Sc. xylosifermentans* and *Sc. coipomoensis*, but these were minimal compared to the activities with NAD^+^ (Fig. 3A). For species that possess NADPH-preferring XRs, the exclusive utilization of NAD^+^ by XDH could potentially hinder the conversion of xylitol to xylulose under oxygen-limiting conditions, resulting in xylitol accumulation due to the previously proposed redox imbalance hypothesis [35, 36]. Nonetheless, the xylitol bottleneck observed in *Sc. xylosifermentans* with high aeration seems to require a different explanation because our enzyme assays suggested that its pathway was more redox balanced due to its NADH-utilizing XR.

### Expression of genes related to xylose metabolism is highly affected by oxygen

To determine whether oxygen levels (SF compared to BF) affected gene expression in a redox-independent way that could impact xylose-to-ethanol flux, we sequenced mRNA by taking samples in mid-log phase from the same species used in the enzyme assays. After analyzing the data and filtering the results with specific thresholds (see Methods), we identified 2263, 2546, 2111, and 741 differentially expressed genes (DEGs) for *Sp. passalidarum*, *Sc. xylosifermentans*, *Sc. coipomoensis*, and *Sc. amazonensis*, respectively. This differential expression analysis showed that xylose metabolism was highly affected by oxygenation. The genes encoding the first three steps of the xylose catabolism pathway were upregulated in SF (relative to BF) for all species, except for *Sc. coipomoensis XYL2*, which was not differentially expressed. For *Sp. passalidarum*, *XYL1.1* and *XYL2.2* were downregulated in SF compared to BF, but *XYL1.2* (the homolog that encodes the XR with NADH affinity) and *XYL2.1* were upregulated. Interestingly, *XYL2* was among the 15 most upregulated genes in SF in *Sc. xylosifermentans*.

**Fig. 4:**
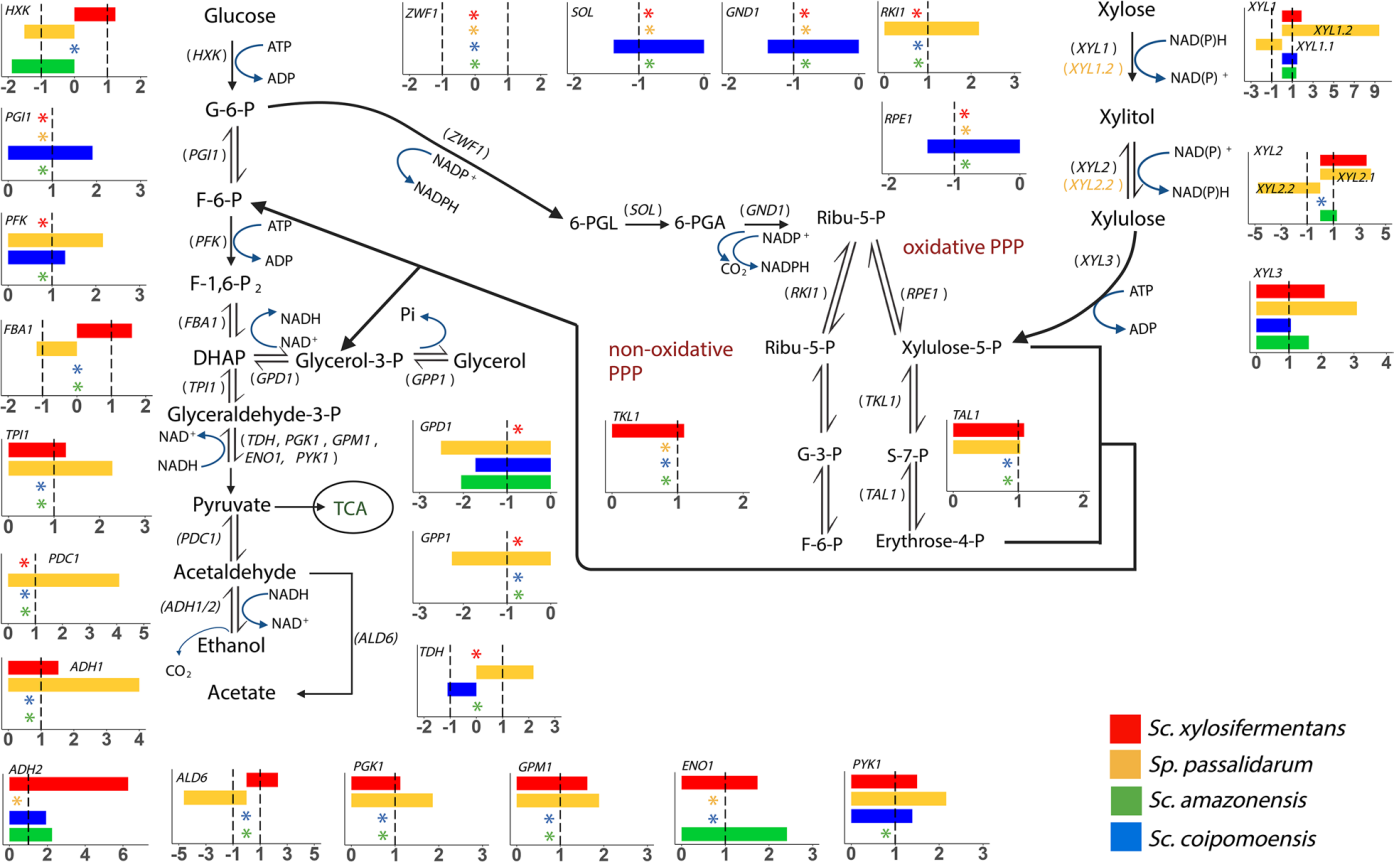
Several genes related to xylose fermentation were upregulated only for ethanol producers at lower oxygenation. Xylose metabolism and related pathways. Dashed lines correspond to the < -1 and > 1 confidence intervals for the log_2_-fold change from RNA-Seq analysis (moderate aeration/high aeration). Asterisks indicate genes that did not exhibit differential expression. Positive and negative values indicate upregulated and downregulated genes, respectively, and the colors are associated with the species as shown in the right side of the figure. Protein products encoded by each gene: *HXK*—hexokinase;* PGI1*—phosphoglucose isomerase;* PFK*—phosphofructokinase;* FBA1*—fructose 1,6-bisphosphate aldolase;* TPI1*—triose phosphate isomerase;* TDH*—glyceraldehyde-3-phosphate dehydrogenase;* PGK1*—3-phosphoglycerate kinase;* GPM1*—phosphoglycerate mutase;* ENO*—enolase*; PYK1*—pyruvate kinase*; PDC1*—pyruvate decarboxylase*, ADH1/2*—alcohol dehydrogenase;* ALD6*—aldehyde dehydrogenase; *GPD1*—glycerol-3-phosphate dehydrogenase;* GPP1*—glycerol-3-phosphate phosphatase;* ZWF1*—glucose-6-phosphate dehydrogenase;* SOL*—6-phosphogluconolactonase;* GND1*—6-phosphogluconate dehydrogenase;* RKI1*—ribose-5-phosphate ketol-isomerase;* RPE1*—d-ribulose-5-phosphate 3-epimerase;* TKL1*—transketolase;* TAL1*—transaldolase;* XYL1*—xylose reductase;* XYL2*—xylitol dehydrogenase*,* and *XYL3*—xylulokinase. Created with BioRender.com [75] with license number EN2651FNZW

**Fig. 5:**
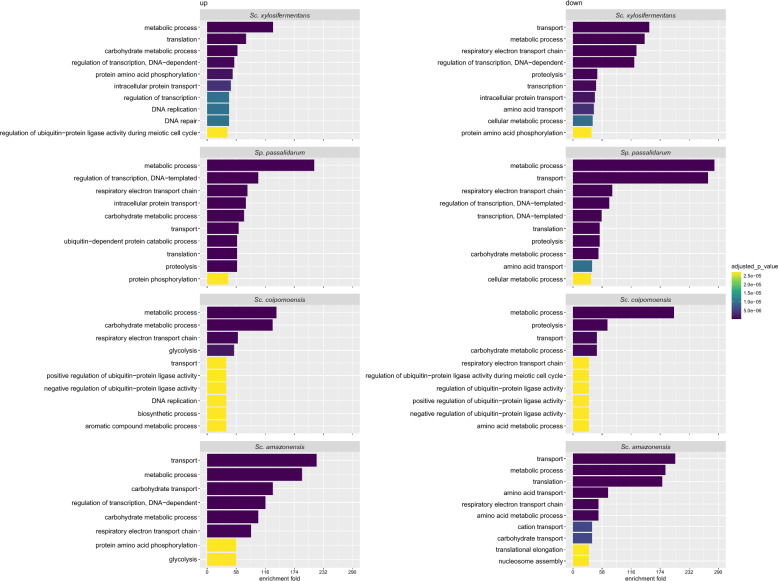
Metabolic and carbohydrate metabolic processes are highly affected by oxygenation. Enrichment analysis for *Sc. xylosifermentans*, *Sc. coipomoensis*, *Sc. amazonensis*, and *Sp. passalidarum*. On the right are the biological processes enriched in the upregulated gene list, while those the left side are enriched in the downregulated gene list (moderate aeration/high aeration)

Figure 4 shows the DEGs in key pathways from the comparison of SF and BF. Genes related to the non-oxidative shunt of the PPP, such as *TKL1* and *TAL1*, were also upregulated in SF in ethanol producers under moderate aeration, but there were no DEGs for xylitol producers related to those genes. From the oxidative portion of the PPP, which is not essential for xylose catabolism, some genes, such as *SOL1*, *GND1*, and *RPE1*, were downregulated in *Sc. coipomoensis*. Outside of the PPP, genes that include *TPI1*, *PDC1*, *PGK1*, *GPM1*, and *ADH1* were also upregulated in SF only in ethanol producers. *ADH1* and *ADH2* are two genes related to ethanol production and consumption in *Sc. stipitis* [37, 38]. While *ADH1* was differentially expressed only in ethanol producers, *ADH2* was also upregulated in xylitol producers and was the highest DEG in *Sc. xylosifermentans*. Although the ethanol producers upregulated key genes related to xylose metabolism in SF, the genes that were upregulated or downregulated in the four species were enriched in biological processes, such as metabolic and carbohydrate metabolic processes (Fig. 5). These biological processes, along with electron transport, likely have important roles that affect the observed phenotypes, suggesting that relevant pathways and genes related to metabolism were highly affected in the conditions tested.

### *XYL* genes from *Scheffersomyces xylosifermentans* enhance anaerobic xylose fermentation by *Saccharomyces cerevisiae*

**Fig. 6:**
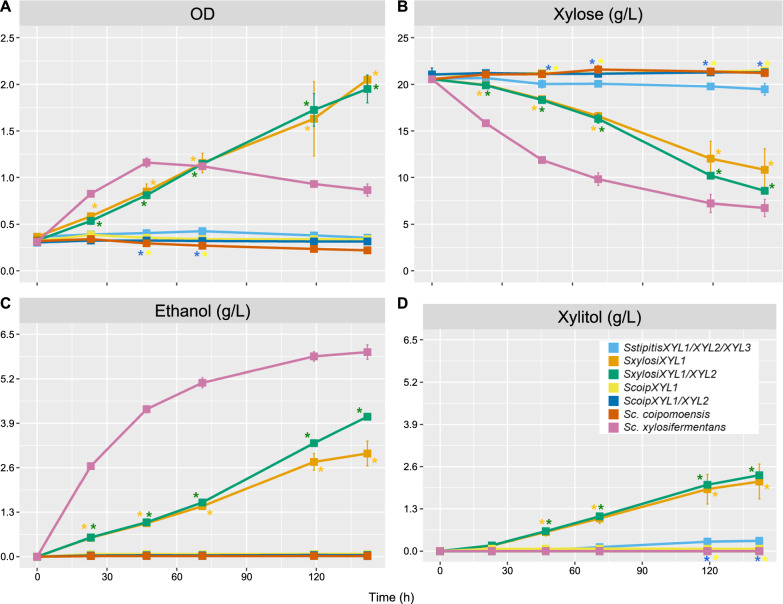
*Saccharomyces cerevisiae* with *XYL* genes from *Scheffersomyces xylosifermentans* ferments xylose anaerobically. Growth curves of *Sc. xylosifermentans*, *Sc. coipomoensis*, and *Sc. cerevisiae* strains carrying different *XYL* genes under anoxic conditions **A** OD, **B** xylose consumption, **C** ethanol production, and **D** xylitol accumulation. Error bars indicate the standard deviation from the three biological replicates. Asterisks denote significant differences related to *S. cerevisiae* + *SstipitisXYL1*/*XYL2*/*XYL3*). *SxylosiXYL1* and *SxylosiXYL1*/*XYL2*—*XYL* genes from *Sc. xylosifermentans*; *ScoipXYL1* and *ScoipXYL1*/*XYL2*—*XYL* genes from *Sc. coipomoensis*; and *SstipitisXYL1*/*XYL2*/*XYL3*—*XYL* genes from *Sc. stipitis*

Our phenotypic results showed that *Sc. xylosifermentans* rapidly fermented xylose to ethanol, while *Sc. coipomoensis* accumulated high titers of xylitol. To test the possibility that the XR and XDH enzymes from *Sc. xylosifermentans* would enable greater xylose fermentation than those of *Sc. coipomoensis* enzymes, we used CRISPR-Cas9 to re-engineer a strain of *S. cerevisiae* that expresses *Sc. stipitis XYL1*, *XYL2*, and *XYL3*. *SstipitisXYL1* and *SstipitisXYL2* were replaced with the corresponding genes from *Sc. xylosifermentans* (*SxylosiXYL1* and *SxylosiXYL2*) and *Sc. coipomoensis* (*ScoipXYL1* and *ScoipXYL2*). We hypothesized that the modified *S. cerevisiae* would grow faster anaerobically with *Sc. xylosifermentans XYL1* because this XR enzyme lacked a cofactor preference (Fig. 3). In contrast, this would not be expected for the strain with *XYL1* from *Sc. coipomoensis* or for the original parental strain containing *SstipitisXYL1*. Indeed, the strain expressing *SxylosiXYL1* and *SxylosiXYL1*/*XYL2* grew rapidly under anaerobic conditions compared to the strain *SstipitisXYL1*/*XYL2/XYL3* (*P* < 0.05), while the strains expressing *ScoipXYL1* alone or *ScoipXYL1*/*XYL2* presented an inferior growth profile (*P* < 0.05) to the strain containing *SstipitisXYL1*/*XYL2/XYL3* (Fig. 6A). *Sc. stipitis* showed modest anaerobic growth with very limited production of ethanol or accumulation of intermediates (Additional file 3). This result is interesting because the *Sc. stipitis* XR can use both NADH and NADPH, but it has a preference for the latter [39]. Surprisingly, *Sc. xylosifermentans* grew anaerobically and produced ethanol, but it did not excrete xylitol (Fig. 6A–D). Along with the other species and engineered strains, it also did not anaerobically produce glycerol, which is produced by *S. cerevisiae* under anoxic conditions to alleviate the cytosolic redox balance [40]. Even though the replacement of *Sc. stipitis XYL* genes with *SxylosiXYL1/XYL2* improved xylose fermentation to ethanol by *S. cerevisiae* and enabled anaerobic growth, the mutants still excreted xylitol (Fig. 6D). This result contrasts what we observed natively with *Sc. xylosifermentans,* which did not excrete xylitol in anoxic conditions.

Despite employing ATP, instead of NAD(P)H, as a cofactor for the conversion of xylulose to xylulose-5-P, the xylulokinase enzyme encoded by *XYL3* might contribute to xylitol accumulation due to the reversible nature of the previous reaction that generates xylulose from xylitol [41] (Fig. 4). We hypothesized that *XYL3* could also affect xylitol accumulation, so we also engineered a strain of *S. cerevisiae* that expressed *Sc. xylosifermentans XYL3*. Although this strain still accumulated xylitol, ethanol production, xylose consumption, and growth by *S. cerevisiae* with *SxylosiXYL1/XYL2/XYL3* were generally slightly improved compared to the strains carrying *SxylosiXYL1* and *SxylosiXYL1/XYL2* (*P* < 0.05), which suggests that this gene is also more active than *Sc. stipitis XYL3* (Additional file 3).

Finally, we confirmed that the XR and XDH enzymes maintained their predicted co-factor preferences when expressed *in S. cerevisiae* by performing enzymatic assays using whole-cell lysates from the *S. cerevisiae* strains engineered with *SxylosiXYL1*/*SxylosiXYL2* or *ScoipXYL1*/*ScoipXYL2*. Although the activities were lower compared to the native activities of XR in the donor species, XR encoded by *SxylosiXYL1* in strain GLBRCY1847 (NADH: 1.97 ± 0.01 U mg^−1^; NADPH: 2.15 ± 0.15 U mg^−1^ *P* > 0.05) still had fairly equal preferences between cofactors, while the XR encoded by *ScoipXYL1* in GLBRCY1850 showed a preference for NADPH (NADH: 0.63 ± 0.05 U mg^−1^; NADPH: 1.18 ± 0.03 U mg^−1^, *P* < 0.05). Xylitol dehydrogenase activity was high for NAD^+^ in both GLBRCY1847 (6.69 ± 0.29 U mg^−1^) and GLBRCY1850 (4.24 ± 0.68 U mg^−1^), and no activity was detected with NADP^+^.

## Discussion

To better understand the abilities of yeasts from the order Serinales to metabolize xylose, we quantified the metabolites produced by several species cultured in rich medium with xylose as the primary carbon source under high and moderate aeration conditions. Ethanol producers and xylitol producers were both identified. Most of the yeasts tested fermented xylose, but fewer than half primarily produced ethanol under moderate aeration. Instead, most yeasts mainly produced xylitol in the conditions tested. Xylitol, an intermediate of xylose catabolism, is excreted by several species when a bottleneck in oxidation to xylulose occurs. Accumulation of xylitol in the extracellular medium was believed to occur due to the different XR and XDH cofactor requirements, which leads to cofactor imbalance under oxygen-limited (including moderate aeration) or anoxic conditions [15, 42–44]. Under these conditions, NADH accumulates, and NAD^+^ levels fall. Here, we unexpectedly observed that several species accumulated xylitol when sufficient oxygen was available, including species that were among the best ethanol producers in moderate oxygen-limiting conditions (see Figs. 1 and 2, Additional file 1). This result strongly suggests that the different cofactor requirements by XR and XDH are not the sole issue limiting xylose fermentation.

An important question raised by our fermentation experiments was why the top xylose-fermenting species, *Sc. xylosifermentans*, showed similar or better xylose fermentation than that of *Sp. passalidarum* in moderate aeration, even though the former has a single homolog of *XYL1*, while the latter has two homologs. Enzymatic assays were conducted to determine the activities and cofactor preferences of XR and XDH in each condition for key species. Assuming that redox imbalance primarily impacted xylose fermentation, our initial hypothesis was that the enzyme XR from species with high metabolic activity might present the same features as the XR encoded by *XYL1.2* from *Sp. passalidarum*. In contrast, our findings revealed that the enzymes of all the four species could use both NADH and NADPH as cofactors (Fig. 3A), which was previously reported for *Sc. stipitis* [39]. The *Sc. coipomoensis* XR showed significant activity with NADH under moderate and high aeration conditions, but it still had a preference for NADPH. An NADPH-dependent XR was also present in *Sc. amazonensis*, a species that also accumulated xylitol. Surprisingly, the XR of *Sc. xylosifermentans* did not have a cofactor preference. In this species, the lack of cofactor preference seems to naturally enhance flux for the second reaction of the xylose metabolism due to the oxidation of NADH to NAD^+^, which is the cofactor used in the preceding step. Our results are consistent with a previous observation in which high aeration caused a switch in cofactor usage from NADH to NADPH for XR from *Sp. passalidarum* NRRL Y-27907 [23], and our transcriptome data showed that this switch likely occurs because the two paralogous *XYL1* genes have different levels of expression under moderate and high aeration conditions (Fig. 6). A similar result was reported by Cadete et al. [15], in which *XYL1.2* was 15–16-fold more highly expressed than *XYL1.1* in more extreme oxygen-limiting conditions compared to moderate aeration condition. Collectively, these results suggest that *XYL1.2* expression increases as the availability of oxygen decreases*.* Thus, *Sp. passalidarum* may use its second homolog of *XYL1*, which encodes the NADH-preferring XR, to limit redox imbalance in low oxygen levels. Rapid consumption of xylose and high yields of ethanol were not observed for other species that harbor multiple paralogs, such as *C. intermedia,* which was reported to have a NADH-dependent XR [19]. Thus, *Sp. passalidarum* is unique among the species tested.

To elucidate the effect of oxygen on the expression profile of genes involved in the first steps of xylose metabolism and the PPP, we determined their relative gene expression in *Sc. amazonensis*, *Sc. coipomoensis*, *Sc. xylosifermentans,* and *Sp. passalidarum* under moderate and high aeration conditions. The genes directly involved in xylose metabolism (*XYL1*, *XYL2*, and *XYL3*) were all upregulated with moderate aeration (Fig. 4) for *Sc. xylosifermentans*, which is consistent with the higher XR and XDH activities observed in in vitro enzyme assays (Fig. 3). Indeed, *Sc. xylosifermentans XYL2* was among the 15 most upregulated genes under oxygen limitation, the condition in which this species presented high levels of xylose consumption. Xylitol to xylulose conversion by XDH enzymes has been proposed to be the limiting step for xylose fermentation, and high expression and codon optimization of *XYL2* may be necessary for efficient xylose conversion [16, 45, 46]. Even small expression changes may be associated with large phenotypic effects [47] and, in the case of *Sc. xylosifermentans, XYL2* expression decreased significantly under high aeration. The xylitol accumulation by this species in this condition is likely in part due to this reduction in *XYL2* expression, rather than the cofactor imbalances postulated for some other species.

Based on what is known about *S. cerevisiae* metabolism, the production of glycerol by *Sp. passalidarum* in high aeration is difficult to explain, especially considering the large amount it produced under high aeration. Glycerol can maintain redox balance in the absence of oxygen when ethanol flux is overloaded in *S. cerevisiae* [40]. Strategies to increase glycerol production by yeasts include cutting off or attenuating ethanol production, shifting the NAD^+^/NADH ratio to increase the amount of NADH available, and overexpression of the glycerol 3-phosphate dehydrogenase encoded by *GPD1* [48–50]. The downregulation of *PDC1* and *ADH1*, which encode pyruvate decarboxylase and alcohol dehydrogenase, respectively, in *Sp. passalidarum* and the upregulation of *GPD1* and *GPP1* (encoding glycerol-3-phosphate phosphatase) under high aeration suggest a mechanism for the elevated glycerol production by this species in this condition.

Genes from the PPP, such as *TKL1*, *TAL1*, and *RKI1*, were also upregulated under moderate oxygen-limited condition for *Sc. xylosifermentans* (*TKL1* and *TAL1*) and *Sp. passalidarum* (*TKL1* and *RKI1*). Overexpression of those genes in engineered strains of *S. cerevisiae* improved the growth rate on xylose and the ethanol yield [51, 52]. *ADH1* and *ADH2* are responsible for ethanol production and consumption in *Sc. stipitis*, and when the availability of oxygen becomes limited, the expression of *ADH* genes, especially *ADH2*, increases [37, 53]. While *ADH1* was upregulated in the ethanol-producing yeasts under moderate aeration, *ADH2* was the most upregulated gene for *Sc. xylosifermentans*; *ADH2* was also differentially expressed in *Sc. coipomoensis* and *Sc. amazonensis*, but not *Sp. passalidarum* (Fig. 4). In addition to the genes required for xylose metabolism, the high expression of the PPP and ethanol pathway in *Sp. passalidarum* and *Sc. xylosifermentans* might be related to the greater xylose metabolism by these species under moderate aeration.

Next, we genetically modified *S. cerevisiae* by the chromosomal integration of *XYL* genes from *Sc. xylosifermentans* and *Sc. coipomoensis*. Given the inability of *S. cerevisiae* to metabolize xylose, any observed growth must be attributed to the heterologously and constitutively expressed enzymes, which allowed us to investigate how redox balance influences anaerobic fermentation of xylose into ethanol in this model system. With the rest of the XYL pathway coming from *Sc. stipitis*, *S. cerevisiae* could grow in anaerobic conditions with *SxylosiXYL1* and *SxylosiXYL1/XYL2*, but not with *ScoipXYL1*, *ScoipXYL1/XYL2*, or the *Sc. stipitis* pathway alone (Fig. 6). This result suggests that cofactor preference has important effects in anoxic conditions, at least when the pathway is constitutively expressed (Fig. 6). *S. cerevisiae* strains containing *SxylosiXYL1* and *SxylosiXYL1*/*XYL2* produced ethanol, but they also accumulated xylitol. Swapping *SstipitisXYL3* with *SxylosiXYL3* further enhanced ethanol production but only slightly relieved xylitol accumulation, so we hypothesize that other downstream and interacting native metabolic pathways in *Sc. xylosifermentans* may facilitate its xylitol-to-ethanol flux. Nonetheless, this experiment was essential to test the redox balance hypothesis, and it unexpectedly revealed that *Sc. xylosifermentans* was able to grow under anaerobic conditions, which are used in numerous industrial applications.

## Conclusions

We tested 30 species belonging to the order Serinales for xylose fermentation with a focus on the genera *Spathaspora* and *Scheffersomyces* and their close relatives. Collectively, our data reveal that xylose metabolism in the Serinales is highly plastic and oxygen-dependent. Under high aeration conditions, several species switched from ethanol production to xylitol accumulation. This switch was generally accompanied by a decrease in enzyme activity and expression of genes related to xylose catabolism. While we find a global effect of oxygen availability on xylose metabolism, our data support the hypothesis that xylitol accumulation results from redox imbalance generated by differential cofactor preferences for XR and XDH in some species, but they also point to a novel role for oxygen-responsive gene regulation in other species that accumulate xylitol under high aeration, especially *Sc. xylosifermentans*. Among the species tested, *Sc. xylosifermentans* is also remarkable for its high xylose consumption and ethanol formation under moderate aeration and even in anaerobic conditions, a phenomenon not previously noted for any xylose-fermenting yeast species. This species may be a novel source of potential genes that can be expressed in industrial microbes, such as *S. cerevisiae,* for biofuel production from lignocellulosic feedstocks. Alternatively, *Sc. xylosifermentans* could be subjected to adaptive laboratory evolution or genetic modification to enhance its native potential and transform it into an industrial organism.

## Methods

### Yeast strains and growth experiment conditions

*Candida blattae*, *C. intermedia, Me. caribbica, Me. carpophila*, *Me. guilliermondii*, and 24 strains of *Scheffersomyces* and *Spathaspora* species (see Additional file 4) were obtained from the USDA Agricultural Research Service (ARS) NRRL Culture Collection in Peoria, Illinois, USA; Collection of Microorganisms and Cells of Universidade Federal de Minas Gerais, Belo Horizonte, Minas Gerais, Brazil; and CBS Yeast Collection of the Westerdijk Fungal Biodiversity Institute, Utrecht, the Netherlands.

*Saccharomyces cerevisiae* strain GLBRCY38 was generated from a haploid spore from the diploid GLBRCY2A strain [54] containing an integrated DNA cassette to express constitutively *Sc. stipitis XYL1, XYL2,* and *XYL3* genes from the *HO* locus [18]. GLBRCY2A was sporulated, and individual tetrads were dissected as previously described [55]. Haploid spores were verified for the *XYL* cassette by PCR and selection for the *kanMX* resistance marker. One spore containing the *XYL* cassette, GLBRCY25A, was selected, and all *XYL* gene sequences were fully confirmed for accuracy by PCR and Sanger sequencing. The *kanMX* marker was excised [56] to generate the GLBRCY38 strain. We used CRISPR-Cas9 to swap the genes *XYL1*, *XYL2*, and/or *XYL3* to the corresponding genes from *Sc. xylosifermentans* and *Sc. coipomoensis* in the strain GLBRCY38 with gRNA expression plasmid (pXIPHOS) [57, 58] targeting *XYL1* (CAACAGCCAAAACCCACGGC), *XYL2* (CTTAACCAAGAAATCTTCGG), and/or *XYL3* (TGCCTCCCCACAACCCGAGG). sgRNAs were designed using CRISpy-pop [59]. Multiple swaps were performed sequentially. Transformation of yeast strains was done using the lithium acetate/PEG-4000/carrier DNA method [60]. After the transformation and recovery, colonies were selected by plating them on YPD agar with 100 mg/L nourseothricin (NAT). All strains were confirmed for gene swaps and antibiotic marker excision by PCR with gene-specific primers or flanking primers. Sanger sequencing of purified PCR products was performed by the University of Wisconsin-Madison Biotechnology Center. The engineered strains used in this study are summarized in Additional file 5.

Strains from all yeast species were initially plated from freezer stocks on 10 g/L yeast extract, 20 g/L peptone, and 2% dextrose (YPD) plates and grown for single colonies. Single colonies of each strain were pre-cultured in 10 mL of YPX medium (1% yeast extract, 2% peptone, 2% xylose) overnight at 30 °C under 200 rpm. Cells were recovered by centrifugation at 2600*g* for 10 min, washed with sterile water, and suspended in the fermentation medium at 0.5 g dry cell weight L^−1^ of cell concentration. To evaluate the performance of the yeasts in different aeration conditions, we used 125-mL shake flasks (SF) for moderate aeration and 250-mL baffled flasks (BF) for high aeration, both containing 50 mL of YPX (1% yeast extract, 2% peptone, and 5% xylose). The dissolved oxygen available was measured by a dissolved oxygen meter (Mettler Toledo F4-Field FiveGo, USA). The flasks were incubated at 30 °C under 200 rpm for 72 h. Cell growth was determined by collecting 1 mL of the culture. The cells were recovered by centrifugation and dried in a speed vacuum concentrator. The ethanol, xylitol, and biomass yields (Yp/s^et^, Yp/s^xy^, Yx/s, g g^−1^), volumetric productivity of ethanol and xylitol (Qp^et^, Qp^xy^, g L^−1^ h^−1^), and consumption of d-xylose were determined as described previously by Cadete et al. [15].

Growth experiments using engineered strains were carried out with 125 mL baffled flasks in an anaerobic chamber for 142 h. *Sc. xylosifermentans*, *Sc. coipomoensis*, and *Sc. stipitis* were also tested under this condition. The pre-culture was done exactly as described above, but for inoculation, we shifted into flasks containing 30 mL YPX media (2% xylose) at a concentration of optical density at λ = 600 nm (OD_600_) = 0.3. Seven-hundred microliters of sample were taken during the experiment for measuring the OD_600_ and for quantifying end-products using high-performance liquid chromatography (HPLC) and refractive index detection (RID) as described previously [61]. Plots were constructed in R v3.6.3 using the RStudio v1.3.1073 platform. We performed Student’s *t* tests to determine whether there were significant differences (*P* < 0.05) between the parental strain and engineered strains.

### Enzyme activities

Yeast species *Sc. xylosifermentans*, *Sc. coipomoensis*, and *Sc. amazonensis* were grown in YPX medium as described above or in YPD for the negative control, using both SF and BF. Engineered strains Y1847 and Y1850 were also tested, but they were grown in 125-mL SF with 30 mL YPX (2% xylose) in aerobic conditions. Cells were harvested at mid-log growth phase, washed with cold sterile water, and extracted with Y-PER® Yeast Protein Extraction Reagent (Thermo Fisher). Protein concentrations from the crude cell extracts were determined by BCA Protein Assay Kit (Thermo Fisher). XR activities were obtained from 250-µL reactions containing 100 mM triethanolamine buffer pH 8, 0.2 mM NADPH or NADH, 0.2 M d-xylose, crude cell extract, and deionized water; XDH activities were obtained from 250 µL reactions containing 100 mM glycine buffer pH 9, 50 mM MgCl_2_, 3 mM NADP^+^ or NADP^+^, 0.2 M xylitol, crude cell extract, and deionized water [15]. Enzyme activities were determined by oxidation or reduction of NADH/NADPH or NAD^+^/NADP^+^, respectively. Reaction mixtures aliquoted into 96-well microtiter plates (Corning® 96 Well Clear Flat Bottom UV-Transparent, Darmstadt, Germany) were placed in Tecan® (Infinite M-1000, Switzerland) at 25ºC for measuring absorbance at 340 nm for 1 h. Standard curves of NADPH and NADH were used to calculate the concentration of the samples. Extracted proteins from the yeast *Sp. passalidarum* were used as positive control, while *S. cerevisiae* 288SC was used as a negative control. In addition, blank measurements with samples lacking either cell lysate or xylose substrate were performed for each sample, and the resulting values were subtracted from the test values. Enzyme activities were determined from three independent biological replicates. The specific activity of each enzyme was estimated by the number of enzyme units per mL divided by the concentration of protein in mg/mL. One unit was defined as the generation of 1 μmol NAD(P)H or NAD(P)^+^ per min. We performed paired Student’s *t* tests to determine whether there were significant differences (*P* < 0.05) between NADH and NADPH usage by XR from each species. Data analyses and plots were performed in R v3.6.3 using the RStudio v1.3.1073 platform.

### Genome extraction, sequencing, assembly, and annotation

Genomic DNA (gDNA) of the species *Sc. xylosifermentans*, *Sc. amazonensis*, and *Sc. coipomoensis* was isolated using a modified phenol:chloroform method [62]. The sequencing was performed at the DOE Joint Genome Institute Standard. Genome sequencing was performed using Pacific Biosciences (PacBio) Multiplexed > 10 kb with Blue Pippin Size Selection (AMPure Beads for *Sc. coipomoensis*). Filtered subread data were processed with the JGI quality control pipeline to remove artifacts. The mitochondrial genome was assembled separately with the circular consensus sequencing (CCS) reads and polished with two rounds of RACON version 1.4.13 [63]. The mitochondria-filtered CCS reads were then assembled with Flye version 2.8.1 [64] to generate an assembly and polished with two rounds of RACON version 1.4.13. The *Sc. coipomoensis* nuclear genome was assembled using Falcon v. 1.8.8 [65], improved with FinisherSC, and polished with Arrow version SMRTLink v5.0.1.9578 [66]. Ribosomal DNA (rDNA) was assembled separately from a subset of CCS reads identified using kmer matching against the UNITE database with BBTools (http://sourceforge.net/projects/bbmap) version 38.79. Matching reads were subsampled to 600,000 bp with BBTools, assembled with Flye version 2.8.1, and polished with two rounds of RACON version 1.4.13. The eukaryotic internal transcribed spacer (ITS) was identified and extracted from the rDNA assembly using ITSx (Bengtsson-Palme et al., 2013). Results were used to orient and trim the rDNA contig to 100 bp SSU–1 Kb LSU. Contigs less than 1000 bp were excluded. Completeness of the euchromatic portion of the genome assembly was assessed by aligning assembled consensus RNA sequence data with BBTools. Genomes were then annotated using the JGI annotation pipeline [67] (Additional file 6).

### RNA extraction, sequencing, and data analysis

Cells from *Sp. passalidarum*, *Sc. xylosifermentans*, *Sc. coipomoensis*, and *Sc. amazonensis* were grown on YPD agar for 48 h to single colonies, which were each inoculated into 10 mL of YPX medium overnight in a 50-mL glass tube at 30 °C. After pre-culture, aliquots were transferred to 125 mL SF and 250 mL BF, both flasks with 50 mL of YPX, at an initial of inoculum of 0.5 g L^−1^, and incubated at 30 °C under 200 rpm until mid-log phase. RNA was extracted using the acid phenol protocol [68]. Briefly, the total volume of the cultures was centrifuged with 5% phenol and 95% EtOH, and it was flash-frozen in a dry ice-ethanol bath. Cells were resuspended in TES lysis buffer (10 mM Tris, 10 mM EDTA, 0.5% SDS in water) plus PVP40, acid-washed beads (Sigma #G8772), and one volume of saturated acid phenol (IBI Scientific). Lysates were incubated at 65 °C for 1 h with vortexing every 15 min. Then, the lysates were extracted with 1 volume of acid phenol each and once with 1 volume of chloroform. The aqueous phase of the final chloroform extraction was removed and added to a solution consisting of 2.5 volumes of 95%–100% ethanol and 0.1 volumes of 3 M sodium acetate, and the tubes were placed at − 80 °C overnight to precipitate the RNA. RNA pellets were then collected by centrifugation, washed twice in 70% ethanol each, and resuspended in RNase-free water. Purified RNA was then treated with DNase I (NEB #EN0521) to remove any residual DNA prior to treatment with the RNA Clean & Concentrator kit (Qiagen #74134). Total RNA yields were quantified with the Qubit RNA Assay Kit (Thermo Fisher).

mRNA library preparation, quantification, and sequencing were performed at the DOE Joint Genome Institute. Paired-end libraries were sequenced on Illumina NovaSeq S4 (2 × 151). Raw FASTQ file reads were trimmed to remove adapters and artifact sequences using Trimmomatic version 0.30 [69]. The filtered reads of each species were aligned to their respective reference genome with Bowtie2 version 2.4.5 [70] with average mapping rates of 99.7, 98.8, 98.8, and 98.7% for *Sp. passalidarum*, *Sc. xylosifermentans*, *Sc. coipomoensis*, and *Sc. amazonensis*, respectively. featureCounts [71] was used to generate the raw gene counts. Raw sequencing reads were normalized using the reads per kilobase per million mapped reads (RPKM). DEseq2 version 1.38.1 [72] was used to perform quality control analysis and identify significantly differentially expressed genes (DEGs) from pairwise analyses with the raw counts; we used a Benjamini–Hochberg false discovery rate (FDR) of less than 0.05 as a significance threshold and log_2_-fold change > 1 or < − 1 for differentiating between upregulated and downregulated genes, respectively. The enrichment analysis was done by mapping the gene ontology (GO) terms with the gene identifiers, followed by Fisher’s exact and Benjamini-Hochberg-corrected tests using a threshold of 0.05 with SciPy version 1.9.0 [73] and Python Statsmodels version 0.10.2 [74]. The code is available at https://github.com/kathbarros/goea.

For genome annotation, *Sc. xylosifermentans*, *Sc. amazonensis*, and *Sc. coipomoensis* filtered RNASeq reads were also assembled de-novo with Trinity v.2.3.2 using –normalize_reads and –jaccard_clip options [71].

### Supplementary Information

**Additional file 1.** Xylitol and ethanol are the major bioproducts of xylose fermentation by *Scheffersomyces* and *Spathaspora* species. Rates and yields related to the consumption of d-xylose and production of biomass, ethanol, and xylitol under moderate (shake flask—SF) and high (baffled flask—BF) aeration conditions. Data are summarized in Fig. 1.

**Additional file 2.** Species with multiple homologs of *XYL1* accumulate xylitol. Xylose fermentation by closely related species under moderate (shake flask—SF) and high aeration (baffled flask—BF). Error bars indicate the standard deviation from the three independent replicates.

**Additional file 3.** Growth curves of *Sc. stipitis* and *S. cerevisiae* with *Sc. xylosifermentans XYL1*, *XYL2*, and *XYL3* under anoxic conditions. Error bars indicate the standard deviation from the three biological replicates. Asterisks denote significant differences between *S. cerevisiae* + *SstipitisXYL1/XYL2/XYL3*. The letter **a** indicates significant differences between *S. cerevisiae* + *SxylosiXYL1* and *SxylosiXYL1/XYL2/XYL3*, and the letter **b** indicates significant differences between *S. cerevisiae* + *SxylosiXYL1/XYL2* and *SxylosiXYL1/XYL2/XYL3*.

**Additional file 4. **Yeast species used in this study.

**Additional file 5. ***S. cerevisiae* strains used in this study.

**Additional file 6. **Assembly and annotation statistics for the genomes sequenced in this study.

## Data Availability

Supporting data are available in additional files. Raw RNA-Seq reads of *Sc. xylosifermentans* CBS 12540^T^, *Sc. coipomoensis* NRRL Y-17651^T^, and *Sc. amazonensis* UFMG-HMD-26.3^T^ are available on the JGI portal http://genome.jgi.doe.gov and have been deposited to NCBI’s SRA under the BioProject accessions (PRJNA1015965, PRJNA455445, and PRJNA1015964, respectively). The reference genome of *Sp. passalidarum* NRRL Y-27907^T^ is publicly available (Wohlbach et al., 2011). The genome assemblies and annotations of *Sc. xylosifermentans*, *Sc. amazonensis*, and *Sc. coipomoensis* are available from the JGI fungal genome portal MycoCosm [67] (https:/mycocosm.jgi.doe.gov) and have been deposited at DDBJ/EMBL/GenBank under the BioProject accessions PRJNA1015837, PRJNA1015963, and PRJNA460959, respectively. RNA-Seq enrichment analysis code is available at https://github.com/kathbarros/goea.
