# Supplementary material for: Oxygenation influences xylose fermentation and gene expression in the yeast genera Spathaspora and Scheffersomyces
Source: Biotechnol Biofuels Bioprod. 2024 Feb 7;17:20. doi: 10.1186/s13068-024-02467-8 (PMC10848558; doi:10.1186/s13068-024-02467-8)
Supplement: Supplementary file 1 — Additional file 1. Xylitol and ethanol are the major bioproducts of xylose fermentation by Scheffersomyces and Spathaspora species. Rates and yields related to the consumption of d-xylose and production of biomass, ethanol, and xylitol under moderate (shake flask—SF) and high (baffled flask—BF) aeration conditions. Data are summarized in Fig. 1. [file 13068_2024_2467_MOESM1_ESM.docx]

**Additional file 1**: Production of ethanol and xylitol from D-xylose cultures by *Spathaspora* and *Scheffersomyces* species.

| Aeration condition | Yeast species | Xylose consumption (%)* | Biomass  (g L^-1^) | Y_x/s_  (g g^-1^)^†^ | Ethanol  (g L^-1^) | Y_p/s_^et^  (g g^-1^)^†^ | Qp^et^  (g/h L^-1^)^⁑^ | η^et^ (%)^‣^ | Xylitol  (g L^-1^) | Y_p/s_^xyl^ (g g^-1^)^†^ | Qp^xyl^  (g/h L^-1^)^⁑^ | η^xyl^ (%)^‣^ | Time (h)^⁋^ |
| --- | --- | --- | --- | --- | --- | --- | --- | --- | --- | --- | --- | --- | --- |
|  | *Sc. amazonensis* | 99 | 9.90 ± 0.16 | 0.20 | 4.40 ± 0.17 | 0.09 | 0.09 | 18 | 36.1 ± 0.37 | 0.73 | 0.75 | 80 | 48 |
|  | *Sc. coipomoensis* | 99 | 16.2 ± 0.51 | 0.18 | 4.77 ± 0.03 | 0.10 | 0.10 | 19 | 32.1 ± 1.20 | 0.64 | 0.67 | 70 | 48 |
|  | *Sc. cryptocercus* | 99 | 9.01 ± 0.10 | 0.17 | 14.6 ± 0.42 | 0.29 | 0.30 | 57 | 4.40 ± 0.04 | 0.09 | 0.09 | 10 | 48 |
|  | *Sc. illinoinensis* | 99 | 9.89 ± 0.43 | 0.19 | 14.5 ± 0.34 | 0.29 | 0.30 | 56 | 1.77 ± 0.12 | 0.04 | 0.04 | 4 | 48 |
|  | *Sc. insectosa* | 99 | 13.1 ± 0.25 | 0.25 | 8.10 ± 0.13 | 0.16 | 0.11 | 31 | 19.6 ± 0.89 | 0.38 | 0.27 | 61 | 72 |
|  | *Sc. parashehatae* | 99 | 10.1 ± 0.19 | 0.22 | 19.4 ± 0.20 | 0.39 | 0.81 | 78 | 1.39 ± 0.12 | 0.03 | 0.06 | 3 | 24 |
|  | *Sc. quercinus* | 94 | 13.5 ± 0.05 | 0.29 | 4.00 ± 0.02 | 0.08 | 0.08 | 16 | 25.4 ± 0.35 | 0.53 | 0.53 | 58 | 48 |
| Moderate (SF) | *Sc. shehatae* | 99 | 10.2 ± 0.16 | 0.24 | 16.0 ± 0.15 | 0.32 | 0.33 | 63 | 1.52 ± 0.10 | 0.03 | 0.03 | 3 | 48 |
|  | *Sc. stipitis* | 99 | 10.8 ± 0.28 | 0.22 | 15.6 ± 0.31 | 0.31 | 0.33 | 61 | 0.65 ± 0.02 | 0.01 | 0.01 | 1 | 48 |
|  | *Sc. virginianus* | 99 | 7.51 ± 0.11 | 0.13 | 19.1 ± 0.28 | 0.39 | 0.40 | 76 | 1.61 ± 0.06 | 0.03 | 0.03 | 4 | 48 |
|  | *Sc. xylosifermentans* | 99 | 4.90 ± 0.31 | 0.08 | 21.9 ± 0.50 | 0.41 | 0.91 | 82 | 0.37 ± 0.00 | 0.01 | 0.02 | 1 | 24 |
|  | *Sp. arborariae* | 99 | 10.4 ± 0.08 | 0.21 | 17.7 ± 0.20 | 0.36 | 0.37 | 70 | 3.05 ± 0.29 | 0.06 | 0.06 | 7 | 48 |
|  | *Sp. brasiliensis* | 98 | 10.8 ± 0.30 | 0.21 | 5.66 ± 0.06 | 0.12 | 0.08 | 23 | 24.9 ± 0.12 | 0.51 | 0.35 | 55 | 72 |
|  | *Sp. girioi* | 99 | 9.96 ± 0.10 | 0.19 | 5.27 ± 0.12 | 0.11 | 0.07 | 21 | 22.5 ± 0.31 | 0.45 | 0.31 | 49 | 72 |
|  | *Sp. gorwiae* | 47 | 12.2 ± 0.22 | 0.50 | 1.21 ± 0.10 | 0.05 | 0.02 | 10 | 0.00 ± 0.00 | 0.00 | 0.00 | 0 | 72 |
|  | Yeast species | Xylose consumption (%)* | Biomass  (g L^-1^) | Y_x/s_  (g g^-1^)^†^ | Ethanol  (g L^-1^) | Y_p/s_^et^  (g g^-1^)^†^ | Qp^et^  (g/h L^-1^)^⁑^ | η^et^ (%)^‣^ | Xylitol  (g L^-1^) | Y_p/s_^xyl^ (g g^-1^)^†^ | Qp^xyl^  (g/h L^-1^)^⁑^ | η^xyl^ (%)^‣^ | Time (h)^⁋^ |
|  | *Sp. hagerdaliae* | 99 | 10.3 ± 0.50 | 0.20 | 8.60 ± 0.04 | 0.17 | 0.18 | 34 | 16.2 ± 0.41 | 0.32 | 0.22 | 35 | 48 |
|  | *Sp. materiae* | 99 | 8.80 ± 0.20 | 0.17 | 3.54 ± 0.11 | 0.07 | 0.05 | 14 | 33.5 ± 0.25 | 0.68 | 0.47 | 74 | 72 |
| Moderate (SF) | *Sp. passalidarum* | 99 | 8.70 ± 0.23 | 0.15 | 18.9 ± 0.13 | 0.35 | 0.79 | 69 | 0.85 ± 0.01 | 0.02 | 0.04 | 2 | 24 |
|  | *Sp. roraimanensis* | 100 | 7.50 ± 0.05 | 0.16 | 7.55 ± 0.01 | 0.17 | 0.16 | 34 | 16.2 ± 0.22 | 0.38 | 0.34 | 41 | 48 |
|  | *Sp. suhii* | 98 | 9.40 ± 0.31 | 0.18 | 7.08 ± 0.20 | 0.14 | 0.10 | 28 | 19.7 ± 0.17 | 0.40 | 0.27 | 44 | 72 |
|  | *Sp. xylofermentans* | 100 | 12.0 ± 0.20 | 0.23 | 4.22 ± 0.03 | 0.09 | 0.06 | 17 | 25.0 ± 0.23 | 0.51 | 0.35 | 56 | 72 |
|  | *Sc. amazonensis* | 99 | 19.2 ± 1.32 | 0.37 | 0.03 ± 0.00 | 0.00 | 0.00 | 0.1 | 0.68 ± 0.00 | 0.01 | 0.01 | 2 | 72 |
|  | *Sc. coipomoensis* | 99 | 14.0 ± 0.72 | 0.27 | 1.28 ± 0.03 | 0.03 | 0.02 | 5 | 0.30 ± 0.00 | 0.01 | 0.00 | 1 | 72 |
|  | *Sc. cryptocercus* | 99 | 22.7 ± 0.72 | 0.45 | 0.02 ± 0.00 | 0.00 | 0.00 | 0.1 | 10.9 ± 0.08 | 0.22 | 0.15 | 24 | 72 |
|  | *Sc. illinoinensis* | 99 | 19.7 ± 1.45 | 0.38 | 0.03 ± 0.01 | 0.00 | 0.00 | 0.1 | 0.87 ± 0.00 | 0.02 | 0.01 | 2 | 72 |
|  | *Sc. insectosa* | 99 | 15.1 ± 0.19 | 0.29 | 0.13 ± 0.08 | 0.00 | 0.00 | 0.5 | 17.9 ± 0.14 | 0.36 | 0.25 | 39 | 72 |
|  | *Sc. parashehatae* | 99 | 15.6 ± 0.58 | 0.31 | 0.06 ± 0.01 | 0.00 | 0.00 | 0.2 | 8.10 ± 0.20 | 0.16 | 0.11 | 18 | 72 |
| High (BF) | *Sc. quercinus* | 100 | 13.5 ± 0.12 | 0.27 | 0.08 ± 0.04 | 0.00 | 0.00 | 0.3 | 1.99 ± 0.01 | 0.04 | 0.03 | 5 | 72 |
|  | *Sc. shehatae* | 100 | 13.6 ± 0.20 | 0.25 | 0.08 ± 0.01 | 0.00 | 0.00 | 0.3 | 2.32 ± 0.02 | 0.05 | 0.03 | 5 | 72 |
|  | *Sc. stipitis* | 100 | 14.4 ± 0.42 | 0.29 | 0.04 ± 0.00 | 0.00 | 0.00 | 0.2 | 1.50 ± 0.05 | 0.03 | 0.02 | 3 | 72 |
|  | Yeast species | Xylose consumption (%)* | Biomass  (g L^-1^) | Y_x/s_  (g g^-1^)^†^ | Ethanol  (g L^-1^) | Y_p/s_^et^  (g g^-1^)^†^ | Qp^et^  (g/h L^-1^)^⁑^ | η^et^ (%)^‣^ | Xylitol  (g L^-1^) | Y_p/s_^xyl^ (g g^-1^)^†^ | Qp^xyl^  (g/h L^-1^)^⁑^ | η^xyl^ (%)^‣^ | Time (h)^⁋^ |
|  | *Sc. virginianus* | 100 | 16.0 ± 0.70 | 0.32 | 0.12 ± 0.00 | 0.00 | 0.00 | 0.5 | 3.27 ± 0.10 | 0.07 | 0.05 | 7 | 72 |
|  | *Sc. xylosifermentans* | 70 | 15.2 ± 0.91 | 0.43 | 0.12 ± 0.03 | 0.00 | 0.00 | 0.1 | 4.10 ± 0.29 | 0.12 | 0.06 | 13 | 72 |
|  | *Sp. arborariae* | 92 | 19.8 ± 0.72 | 0.05 | 0.09 ± 0.01 | 0.00 | 0.00 | 0.4 | 0.00 ± 0.00 | 0.00 | 0.00 | 0 | 72 |
|  | *Sp. brasiliensis* | 99 | 20.0 ± 0.89 | 0.38 | 0.05 ± 0.00 | 0.00 | 0.00 | 0.2 | 0.00 ± 0.00 | 0.00 | 0.00 | 0 | 72 |
| High (BF) | *Sp. girioi* | 47 | 12.2 ± 0.13 | 0.49 | 0.47 ± 0.02 | 0.02 | 0.01 | 4 | 0.00 ± 0.00 | 0.00 | 0.00 | 0 | 72 |
|  | *Sp. gorwiae* | 93 | 21.7 ± 0.35 | 0.46 | 0.02 ± 0.00 | 0.00 | 0.00 | 0.1 | 3.51 ± 0.03 | 0.08 | 0.05 | 8 | 72 |
|  | *Sp. hagerdaliae* | 99 | 22.7 ± 1.12 | 0.44 | 0.08 ± 0.03 | 0.00 | 0.00 | 0.3 | 1.49 ± 0.29 | 0.03 | 0.02 | 3 | 72 |
|  | *Sp. materiae* | 77 | 11.6 ± 0.25 | 0.29 | 0.02 ± 0.05 | 0.00 | 0.00 | 0.1 | 4.43 ± 0.13 | 0.11 | 0.06 | 13 | 72 |
|  | *Sp. passalidarum* | 100 | 18.3 ± 1.08 | 0.36 | 0.12 ± 0.01 | 0.00 | 0.00 | 0.5 | 0.05 ± 0.01 | 0.00 | 0.00 | 0.1 | 72 |
|  | *Sp. roraimanensis* | 99 | 15.4 ± 0.12 | 0.29 | 0.02 ± 0.00 | 0.00 | 0.00 | 0.1 | 0.19 ± 0.08 | 0.00 | 0.00 | 0.4 | 72 |
|  | *Sp. suhii* | 100 | 15.5 ± 0.42 | 0.30 | 0.02 ± 0.01 | 0.00 | 0.00 | 0.1 | 0.00 ± 0.00 | 0.00 | 0.00 | 0 | 72 |
|  | *Sp. xylofermentans* | 99 | 17.8 ± 0.15 | 0.34 | 0.02 ± 0.00 | 0.00 | 0.00 | 0.1 | 0.66 ± 0.02 | 0.01 | 0.01 | 1 | 72 |

* Xylose consumption (%) – initial D-xylose consumed.

† Y_x/s_ (g g^-1^), Y_p/s_^et^ (g g^-1^) and Y_p/s_^xyl^ (g g^-1^) – biomass, ethanol, and xylitol yield, respectively: correlation between biomass, ethanol, or xylitol (ΔP) produced with sugar (ΔS) consumed.

⁑ Qp^et^ and Qp^xyl^ (g L^-1^ h^-1^) – ethanol productivity: ratio between ethanol concentration (g L^-1^) and time (h); xylitol productivity: ratio between xylitol concentration (g L^-1^) and time (h).

‣ η^et^ and η^xyl^ (%) – conversion efficiency: percentage of the maximum theoretical ethanol or xylitol yield (0.511 g ethanol per g D-xylose and 0.917 g xylitol per g D-xylose).

⁋ Time by which maximum ethanol production (g L^-1^) was reached by each species in shake flasks; by the end of fermentation for baffled flasks - when the species reached the maximum cell concentration.

SF - Shake-flask

BF - Baffled-flask
