# Supplementary figures and images for: Oxygenation influences xylose fermentation and gene expression in the yeast genera Spathaspora and Scheffersomyces
Source: Biotechnol Biofuels Bioprod. 2024 Feb 7;17:20. doi: 10.1186/s13068-024-02467-8 (PMC10848558; doi:10.1186/s13068-024-02467-8)

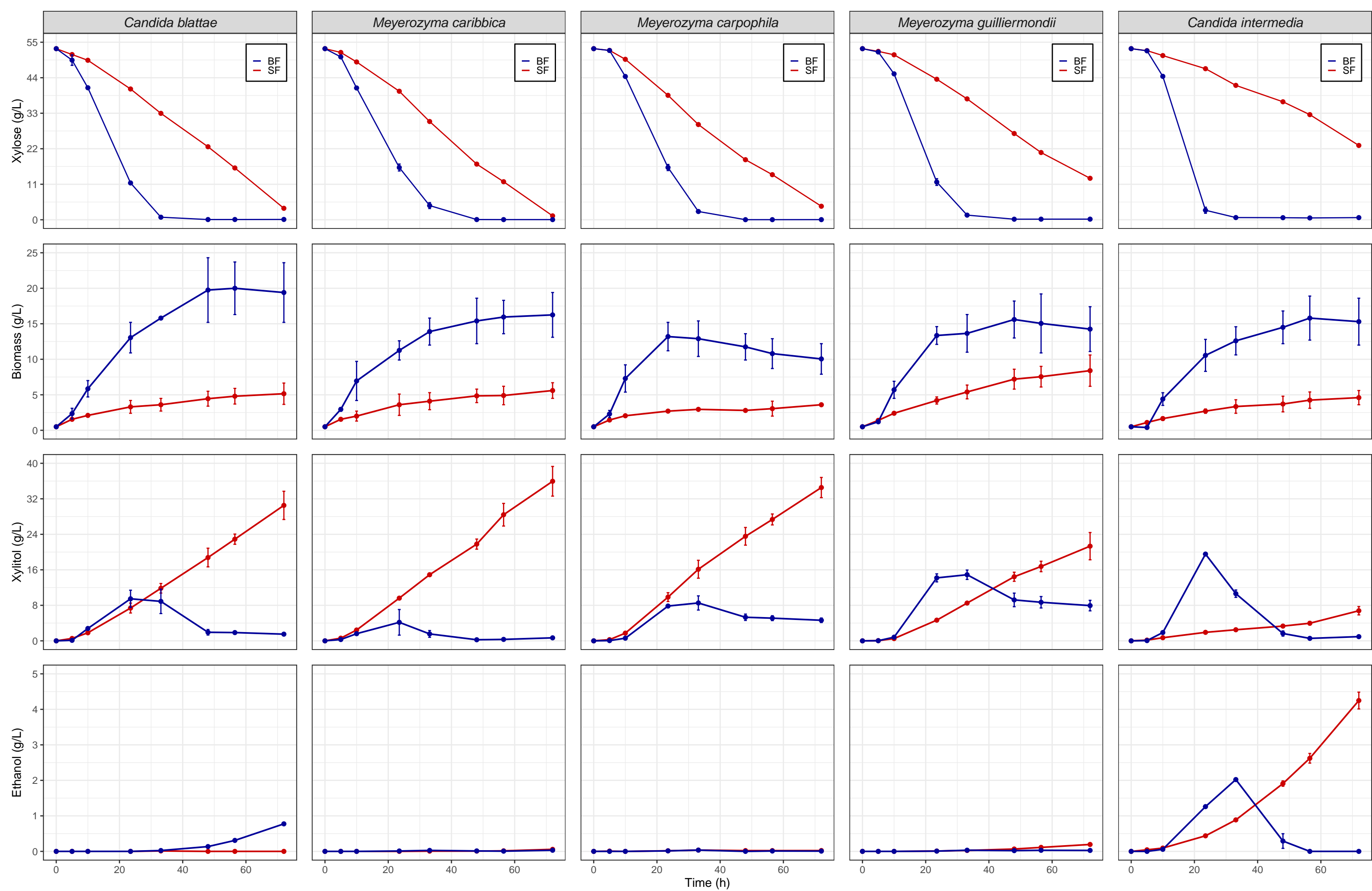

Supplement: Supplementary file 2 — Additional file 2. Species with multiple homologs of XYL1 accumulate xylitol. Xylose fermentation by closely related species under moderate (shake flask—SF) and high aeration (baffled flask—BF). Error bars indicate the standard deviation from the three independent replicates. [file 13068_2024_2467_MOESM2_ESM.pdf]

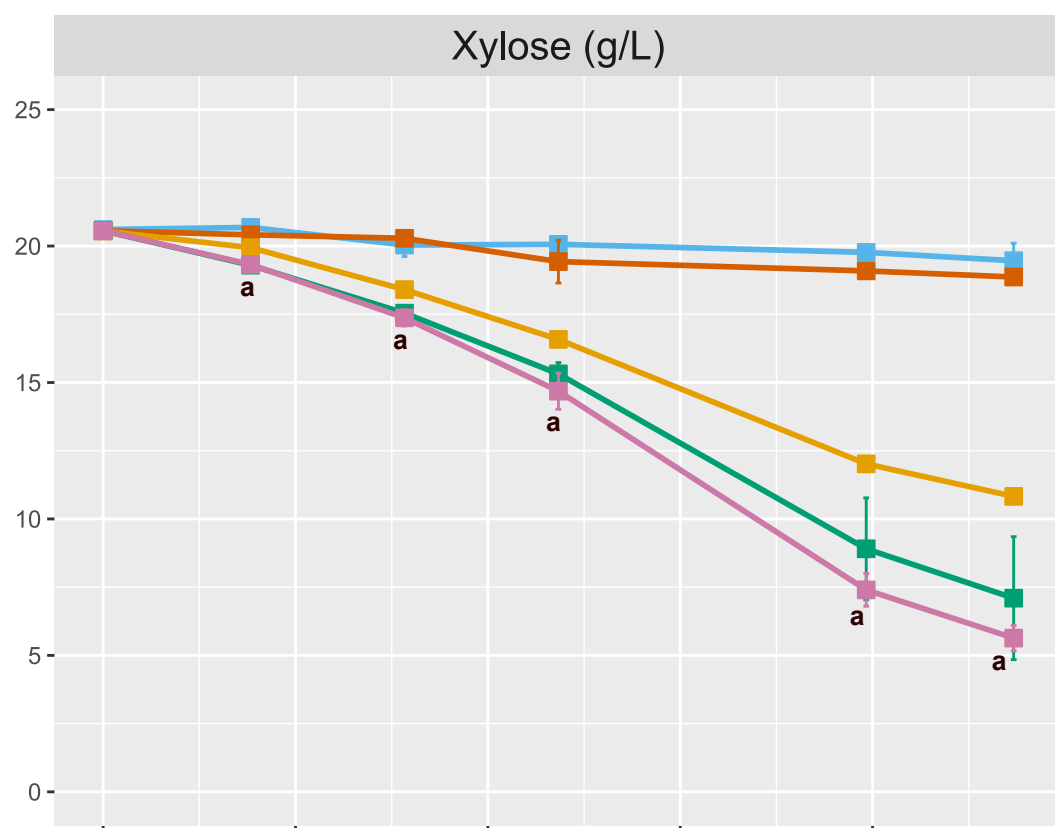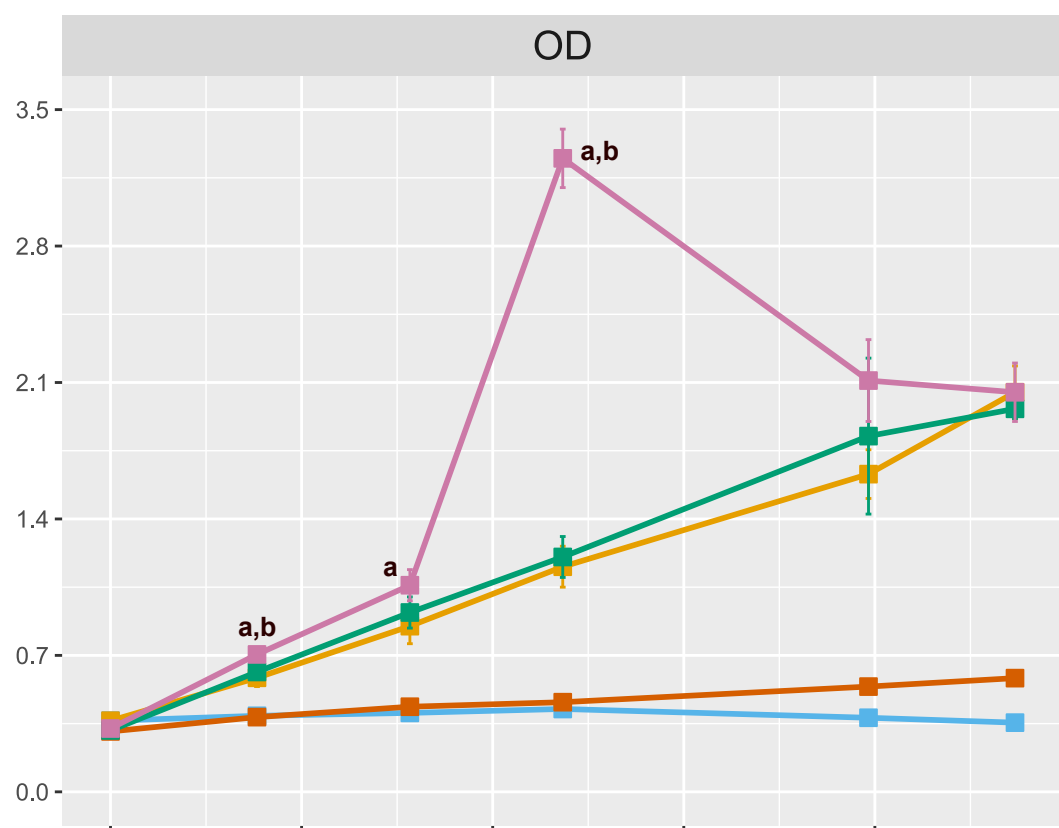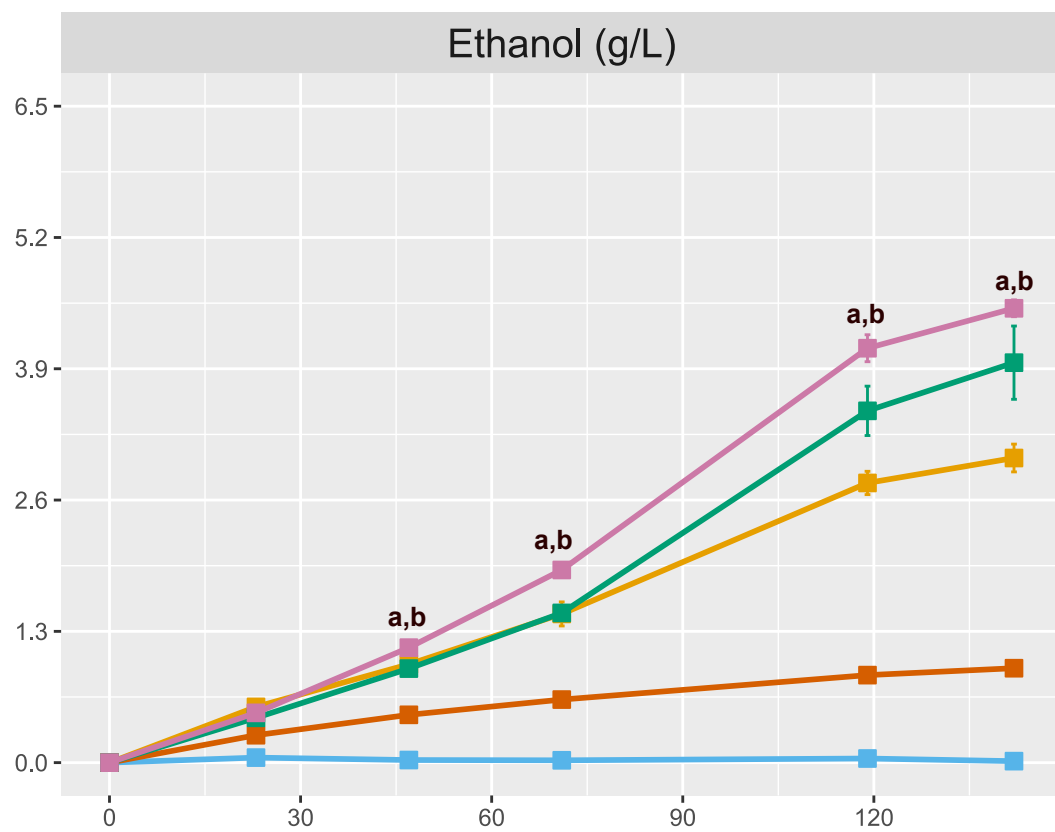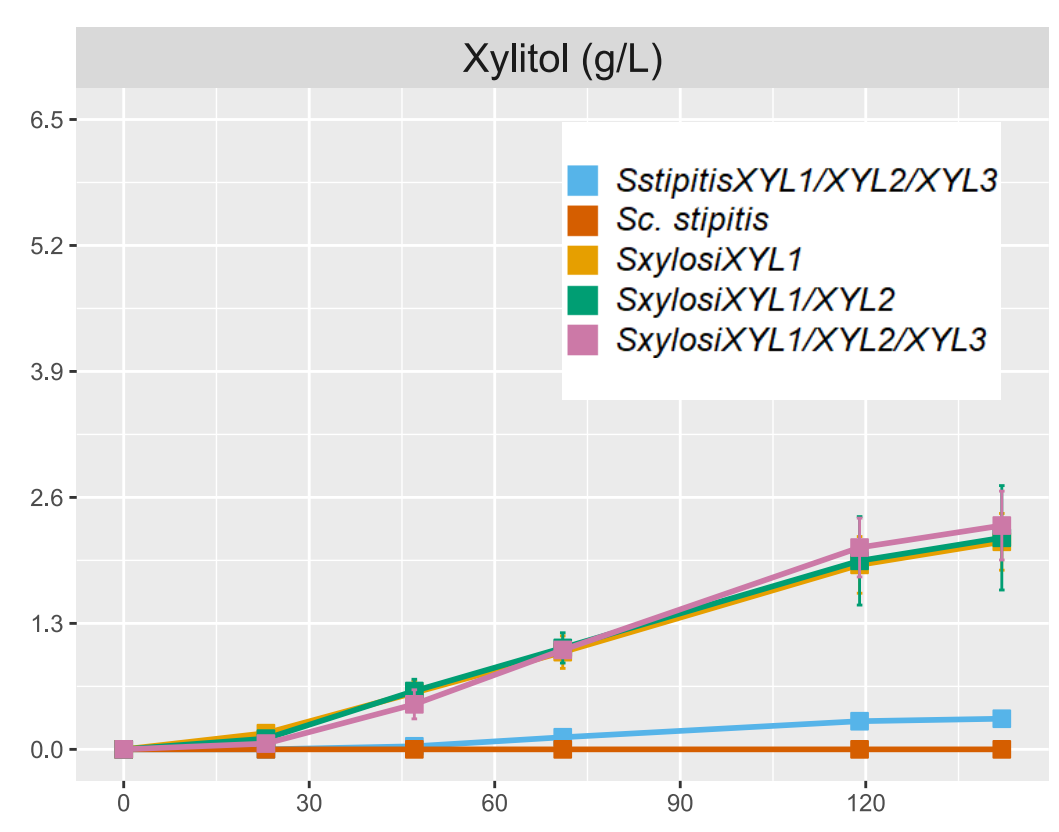

Time (h)

Supplement: Supplementary file 3 — Additional file 3. Growth curves of Sc. stipitis and S. cerevisiae with Sc. xylosifermentans XYL1, XYL2, and XYL3 under anoxic conditions. Error bars indicate the standard deviation from the three biological replicates. Asterisks denote significant differences between S. cerevisiae + SstipitisXYL1/XYL2/XYL3. The letter a indicates significant differences between S. cerevisiae + SxylosiXYL1 and SxylosiXYL1/XYL2/XYL3, and the letter b indicates significant differences between S. cerevisiae + SxylosiXYL1/XYL2 and SxylosiXYL1/XYL2/XYL3. [file 13068_2024_2467_MOESM3_ESM.pdf]
