# Supplementary material for: Oxygenation influences xylose fermentation and gene expression in the yeast genera Spathaspora and Scheffersomyces
Source: Biotechnol Biofuels Bioprod. 2024 Feb 7;17:20. doi: 10.1186/s13068-024-02467-8 (PMC10848558; doi:10.1186/s13068-024-02467-8)
Supplement: Supplementary file 4 — Additional file 4. Yeast species used in this study. [file 13068_2024_2467_MOESM4_ESM.docx]

**Additional file 4**: Yeast species used in this study and their respective isolation sources.

| **Species** | **Strain** | **Source** |
| --- | --- | --- |
| *Candida blattae* | NRRL Y-27698^T^ | insect (1) |
| *Candida intermedia* | CBS 572^T^ | dairy (2) |
| *Meyerozyma caribbica* | CBS 9966^T^ | insect (3) |
| *Meyerozyma carpophila* | CBS 5256^T^ | insect (3) |
| *Meyerozyma guilliermondii* | CBS 2030^T^ | insect frass (3) |
| *Scheffersomyces amazonensis* | UFMG-HMD-26.3^T^ | rotting wood (4) |
| *Scheffersomyces coipomoensis* | NRRL Y-17651^T^ | rotten log (5) |
| *Scheffersomyces cryptocercus* | NRRL Y-48824^T^ | gut of wood cockroach (5) |
| *Scheffersomyces ergatensis* | NRRL Y-17652^T^ | larva of bark beetles (5) |
| *Scheffersomyces gosingicus* | CBS 11433^T^ | soil (6) |
| *Scheffersomyces illinoinensis* | NRRL Y-48827^T^ | rotten wood (7) |
| *Scheffersomyces insectosa* | NRRL Y-12854^T^ | *Leptura maculicornis* (8) |
| *Scheffersomyces parashehatae* | CBS 12535^T^ | larva of *O. disjunctus* (9) |
| *Scheffersomyces quercinus* | NRRL Y-48825^T^ | rotten wood (7) |
| *Scheffersomyces shehatae* | NRRL Y-12858^T^ | soil (7) |
| *Scheffersomyces spartinae* | NRRL Y-7322^T^ | oyster grass (8) |
| *Scheffersomyces stipitis* | NRRL Y-7124^T^ | beetle (8) |
| *Scheffersomyces virginianus* | NRRL Y-48822^T^ | rotten wood (7) |
| *Scheffersomyces xylosifermentans* | CBS 12540^T^ | insect tunnel (9) |
| *Spathaspora arborariae* | UFMG-HMD-32.1 | rotting wood (10) |
| *Spathaspora brasiliensis* | UFMG-HMD-19.3 | rotting wood (11) |
| *Spathaspora girioi* | UFMG-CM-Y302^T^ | rotting wood (12) |
| *Spathaspora gorwiae* | UFMG-CM-Y312^T^ | rotting wood (12) |
| **Species** | **Strain** | **Source** |
| *Spathaspora hagerdaliae* | UFMG-CM-Y303^T^ | rotting wood (12) |
| *Spathaspora materiae* | UFMG-07C151B^T^ | rotting wood (13) |
| *Spathaspora passalidarum* | NRRL Y-27907^T^ | rotting wood (14) |
| *Spathaspora roraimanensis* | UFMG-HMD-23.2^T^ | rotting wood (11) |
| *Spathaspora suhii* | UFMG-CM-Y475^T^ | rotting wood (11) |
| *Spathaspora xylofermentans* | UFMG-CM-Y478^T^ | rotting wood (11) |
|  |  |  |

1. Nguyen NH, Suh SO, Blackwell M. Five novel *Candida* species in insect-associated yeast clades isolated from Neuroptera and other insects. Mycologia. 2007, 99(6):842–58.

2. Lachance MA, Boekhout T, Scorzetti G, Fell JW, Kurtzman CP. Candida Berkhout (1923). In The yeasts 2011 Jan 1 (pp. 987-1278). Elsevier.

3. Vaughan-Martini A, Kurtzman CP, Meyer SA, O’Neill EB. Two new species in the *Pichia guilliermondii* clade: *Pichia caribbica* sp. nov., the ascosporic state of *Candida fermentati*, and *Candida carpophila* comb. nov. FEMS Yeast Research. 2005, 5(45):463–9.

4. Cadete RM, Melo MA, Lopes MR, Pereira GMD, Zilli JE, Vital MJS, et al. *Candida amazonensis* sp. nov., an ascomycetous yeast isolated from rotting wood in the Amazonian forest. International Journal of Systematic and Evolutionary Microbiology. 2012, 62(6):1438–40.

5. Blackwell M, Urbina H, Frank R. *Scheffersomyces cryptocercus*: A new xylose-fermenting yeast associated with the gut of wood roaches and new combinations in the *Sugiyamaella* yeast clade. Mycologia. 2013, 105(3):650–60.

6. Chang CF, Yao CH, Young SS, Limtong S, Kaewwichian R, Srisuk N, et al. *Candida gosingica* sp. nov., an anamorphic ascomycetous yeast closely related to *Scheffersomyces spartinae*. International Journal of Systematic and Evolutionary Microbiology. 2011, 61(3):690-4.

7. Urbina H, Blackwell M. Multilocus phylogenetic study of the *Scheffersomyces* yeast clade and characterization of the N-terminal region of xylose reductase gene. PLoS One. 2012, 7(6):e39128.

8. Suzuki M, Kurtzman CP, stipitis Kurtzman S*. Scheffersomyces* Kurtzman & M. Suzuki (2010). 2010.

9. Suh SO, Houseknecht JL, Gujjari P, Zhou JJ. *Scheffersomyces parashehatae* f.a., sp. nov., *Scheffersomyces xylosifermentans* f.a., sp. nov., *Candida broadrunensis* sp. nov. and *Candida manassasensis* sp. nov., novel yeasts associated with wood-ingesting insects, and their ecological and biofuel implications. International Journal of Systematic and Evolutionary Microbiology. 2013, 63(11):4330–9.

10. Cadete RM, Santos RO, Melo MA, Mouro A, Gonçalves DL, Stambuk BU, et al. *Spathaspora arborariae* sp. nov., a d-xylose-fermenting yeast species isolated from rotting wood in Brazil. FEMS Yeast Research. 2009, 9(8):1338–42.

11. Cadete RM, Melo MA, Zilli JE, Vital MJS, Mouro A, Prompt AH, et al. *Spathaspora brasiliensis* sp. nov., *Spathaspora suhii* sp. nov., *Spathaspora roraimanensis* sp. nov. and *Spathaspora xylofermentans* sp. nov., four novel d-xylose-fermenting yeast species from Brazilian Amazonian forest. Antonie van Leeuwenhoek. 2013, 103(2):421–31.

12. Lopes MR, Morais CG, Kominek J, Cadete RM, Soares MA, Uetanabaro APT, et al. Genomic analysis and D-xylose fermentation of three novel *Spathaspora* species: *Spathaspora girioi* sp. nov., *Spathaspora hagerdaliae* f. a., sp. nov. and *Spathaspora gorwiae* f. a., sp. nov. FEMS Yeast Research. 2016, 16(4):1–12.

13. Barbosa AC, Cadete RM, Gomes FCO, Lachance MA, Rosa CA. *Candida materiae* sp. nov., a yeast species isolated from rotting wood in the Atlantic rain forest. International Journal of Systematic and Evolutionary Microbiology. 2009, 59(8):2104–6.

14. Nguyen NH, Suh SO, Marshall CJ, Blackwell M. Morphological and ecological similarities: wood-boring beetles associated with novel xylose-fermenting yeasts, *Spathaspora passalidarum* gen. sp. nov. and *Candida jeffriesii* sp. nov. Mycological Research. 2006, 110(10):1232–41.
