# Supplementary material for: Oxygenation influences xylose fermentation and gene expression in the yeast genera Spathaspora and Scheffersomyces
Source: Biotechnol Biofuels Bioprod. 2024 Feb 7;17:20. doi: 10.1186/s13068-024-02467-8 (PMC10848558; doi:10.1186/s13068-024-02467-8)
Supplement: Supplementary file 5 — Additional file 5. S. cerevisiae strains used in this study. [file 13068_2024_2467_MOESM5_ESM.docx]

**Additional File 5**: *S. cerevisiae* strains used in this study.

| Modifications to genotype | Strain names | Reference |
| --- | --- | --- |
| NRRL YB-210^MR^ *HO/hoΔ*::*SstipitisXYL1-XYL2-XYL3* | GLBRCY2A | (1)* |
| NRRL YB-210^MR^ *MAT*a spore with *hoΔ*::*SstipitisXYL1-XYL2-XYL3* | GLBRCY38 | This study |
| GLBRCY38 with *SstipitisXYL1Δ*::*SxylosiXYL1* | GLBRCY1840 | This study |
| GLBRCY1840 with *SstipitisXYL2Δ*:: *SxylosiXYL2* | GLBRCY1847 | This study |
| GLBRCY1847 with *SstipitisXYL3Δ*:: *SxylosiXYL3* | GLBRCY1866 | This study |
| GLBRCY38 with *SstipitisXYL1Δ*:: *ScoipXYL1* | GLBRCY1843 | This study |
| GLBRCY1843 with *SstipitisXYL2Δ*:: *ScoipXYL2* | GLBRCY1850 | This study |

* Sato TK, Liu T, Parreiras LS, Williams DL, Wohlbach DJ, Bice BD, et al. Harnessing genetic diversity in *Saccharomyces cerevisiae* for fermentation of xylose in hydrolysates of alkaline hydrogen peroxide-pretreated biomass. Applied and Environmental Microbiology. 2014 Jan 15;80(2):540-54.
